# Long term outcomes and prognostics of visceral leishmaniasis in HIV infected patients with use of pentamidine as secondary prophylaxis based on CD4 level: a prospective cohort study in Ethiopia

**DOI:** 10.1371/journal.pntd.0007132

**Published:** 2019-02-21

**Authors:** Ermias Diro, Tansy Edwards, Koert Ritmeijer, Helina Fikre, Charles Abongomera, Aderajew Kibret, Clélia Bardonneau, Peninah Soipei, Brian Mutinda, Raymond Omollo, Johan van Griensven, Eduard E. Zijlstra, Monique Wasunna, Fabiana Alves, Jorge Alvar, Asrat Hailu, Neal Alexander, Séverine Blesson

**Affiliations:** 1 Leishmaniasis Research and Treatment Centre, University of Gondar, Gondar, Ethiopia; 2 MRC Tropical Epidemiology Group, London School of Hygiene and Tropical Medicine, London, United Kingdom; 3 Médecins sans Frontières, Amsterdam, The Netherlands; 4 Abdurafi Health Centre, Médecins sans Frontières, Ethiopia; 5 Research & Development Department, Drugs for Neglected Diseases *initiative*, Geneva, Switzerland; 6 Drugs for Neglected Diseases *initiative*, Nairobi, Kenya; 7 Institute of Tropical Medicine, Antwerp, Belgium; 8 Department of Microbiology, Immunology, and Parasitology, Addis Ababa University, Addis Ababa, Ethiopia; University of Texas Medical Branch, UNITED STATES

## Abstract

**Background:**

The long-term treatment outcome of visceral leishmaniasis (VL) patients with HIV co-infection is complicated by a high rate of relapse, especially when the CD4 count is low. Although use of secondary prophylaxis is recommended, it is not routinely practiced and data on its effectiveness and safety are limited.

**Methods:**

A prospective cohort study was conducted in Northwest Ethiopia from August 2014 to August 2017 (NCT02011958). HIV-VL patients were followed for up to 12 months. Patients with CD4 cell counts below 200/μL at the end of VL treatment received pentamidine prophylaxis starting one month after parasitological cure, while those with CD4 count ≥200 cells/μL were followed without secondary prophylaxis. Compliance, safety and relapse-free survival, using Kaplan-Meier analysis methods to account for variable time at risk, were summarised. Risk factors for relapse or death were analysed.

**Results:**

Fifty-four HIV patients were followed. The probability of relapse-free survival at one year was 50% (95% confidence interval [CI]: 35–63%): 53% (30–71%) in 22 patients with CD4 ≥200 cells/μL without pentamidine prophylaxis and 46% (26–63%) in 29 with CD4 <200 cells/μL who started pentamidine. Three patients with CD4 <200 cells/μL did not start pentamidine. Amongst those with CD4 ≥200 cells/μL, VL relapse was an independent risk factor for subsequent relapse or death (adjusted rate ratio: 5.42, 95% CI: 1.1–25.8). Except for one case of renal failure which was considered possibly related to pentamidine, there were no drug-related safety concerns.

**Conclusion:**

The relapse-free survival rate for VL patients with HIV was low. Relapse-free survival of patients with CD4 count <200cells/μL given pentamidine secondary prophylaxis appeared to be comparable to patients with a CD4 count ≥200 cells/μL not given prophylaxis. Patients with relapsed VL are at higher risk for subsequent relapse and should be considered a priority for secondary prophylaxis, irrespective of their CD4 count.

## Author summary

Achieving parasitological cure at the end of visceral leishmaniasis (VL) treatment in HIV co-infected patients does not assure definitive cure, as the disease will recur within a year in many patients. In this cohort study, the probability of relapse-free survival at one-year was 50% in all patients. The use of monthly pentamidine infusion for those with lower CD4 counts (<200 cells/μL) at the time of VL cure appeared to result in a comparable relapse-free survival rate to those patients with higher CD4 count (≥200 cells/μL) who did not receive secondary prophylaxis. On the other hand, patients with a history of previous VL treatment (VL relapse) remained at high risk of relapse despite achieving CD4 count ≥200 cells/μL at the end of the VL treatment. While all VL patients with HIV co-infection may benefit from secondary prophylaxis, those with CD4 <200 cells/μL and previous history of treatment should be prioritized for secondary prophylaxis. New modalities for prevention of VL relapse in HIV patients should also be explored.

## Introduction

When visceral leishmaniasis (VL) occurs in HIV patients, it presents several challenges [1]. These include changes in clinical manifestations that may result in delayed diagnosis; changes in immunological response to the infection that affect the performance of diagnostic tools; and poor treatment response, in terms of low initial cure, relapse and mortality, due mainly to the combined effects of both infections causing profound immunosuppression [2,3].

The anti-leishmanial medicines available cannot completely eradicate the *Leishmania* parasites from the body [4]. Even those patients who are declared parasitologically cured at the end of treatment are, in reality, left with some parasites in the tissues that are not undetectable by microscopy [5]. In immunocompetent individuals, these are contained by cell mediated immunity, probably providing some degree of protection. However, in HIV patients, this small number of remaining parasites continues to replicate resulting in relapse of disease [6], which then becomes more difficult to cure. These patients tend to have a persistent infection with flare-ups of clinical disease, described as active chronic disease [7]. Despite repeated treatment courses, such patients remain poorly responsive to treatment and deteriorate in their clinical and immune status. This leads to a high rate of failure to both VL and HIV treatments, and risk of death [8].

Hence, it is logical to introduce a maintenance therapy or secondary prophylaxis for this group of patients, as is done for other opportunistic infections in HIV, to continually suppress the multiplication of the parasite. Although secondary prophylaxis has been recommended in some international guidelines [9,10], this is based on small studies from *L infantum* transmission in Europe [11,12]. In anthroponotic transmission regions like Eastern Africa, patients with persistent *Leishmania* parasites may serve as reservoirs of infection. Their need for repeated treatments with the limited available drugs increases the risk that they will be a source of emergent drug-resistant parasites [13]. Thus, first line drugs used to treat leishmaniasis in the region are not good options for use as secondary prophylaxis for fear of enhancing resistance development.

The use of secondary prophylaxis for VL has not been routinely practiced in high endemic regions such as Northwest Ethiopia. Previous studies have demonstrated that the relapse rate of VL in HIV patients is 60–70% within a year of VL treatment [14,15]. Patients with low CD4 count and previous VL episodes were found to be at the highest risk of relapse. A recent prospective cohort study in Northwest Ethiopia has demonstrated 71% relapse free survival at one year among VL-HIV patients using pentamidine as a secondary prophylaxis for patients with CD4 <200 cells/μL or relapsed VL [16]. This is the only report on secondary prophylaxis for VL in the region. The objective of the current study is to document the long-term treatment outcomes of VL in HIV infected patients, namely the relapse-free survival, and to assess risk factors for relapse or death for up to one year after treatment for VL. Pentamidine was used as secondary prophylaxis for patients with CD4 cell count < 200 cells/μL after VL was successfully treated while those patients with CD4 ≥200 cells/μL were followed without secondary prophylaxis.

## Methods

### Study design

This was a prospective cohort study of parasitologically cured VL patients with HIV co-infection. It followed another randomised clinical trial (NCT02011958), that had a non-comparative design to evaluate the efficacy and safety of two treatment regimens for VL in HIV co-infected patients (AmBisome total dose of 40 mg/kg and Ambisome total dose of 30 mg/kg + miltefosine 100mg/day/28days). In the preceding trial, one or two courses of the allocated treatment were given until patients achieved parasitological cure. One course of treatment was defined as the standard dose/duration of therapy with a specified anti-leishmanial drug e.g. a total dose of 40 mg/Kg of Ambisome monotherapy or 30 mg/kg of Ambisome plus 28-day course of miltefosine regimens. Patients who still had not reached cure after two cycles of VL therapy received rescue treatment at the discretion of the treating physician. At the end of VL therapy, patients who had a negative tissue microscopy result for Leishman-Donovan (LD) bodies were eligible for this prospective cohort study.

### Setting

The study was conducted in Northwest Ethiopia at two large leishmaniasis treatment centres: the Leishmaniasis Research and Treatment Centre at University of Gondar Hospital, supported by Drugs for Neglected Diseases *initiative*; and the Abdurafi Health Centre Médecins Sans Frontières Leishmaniasis Treatment Centre. Both are referral centres for complicated leishmaniasis cases.

Each of these centres treats over 400 VL patients every year, of whom around 15–20% are co-infected with HIV. Most are adult seasonal migrant workers who travel from highland areas to work on large lowland farms where VL is endemic.

### Participants

**Fig 1:**
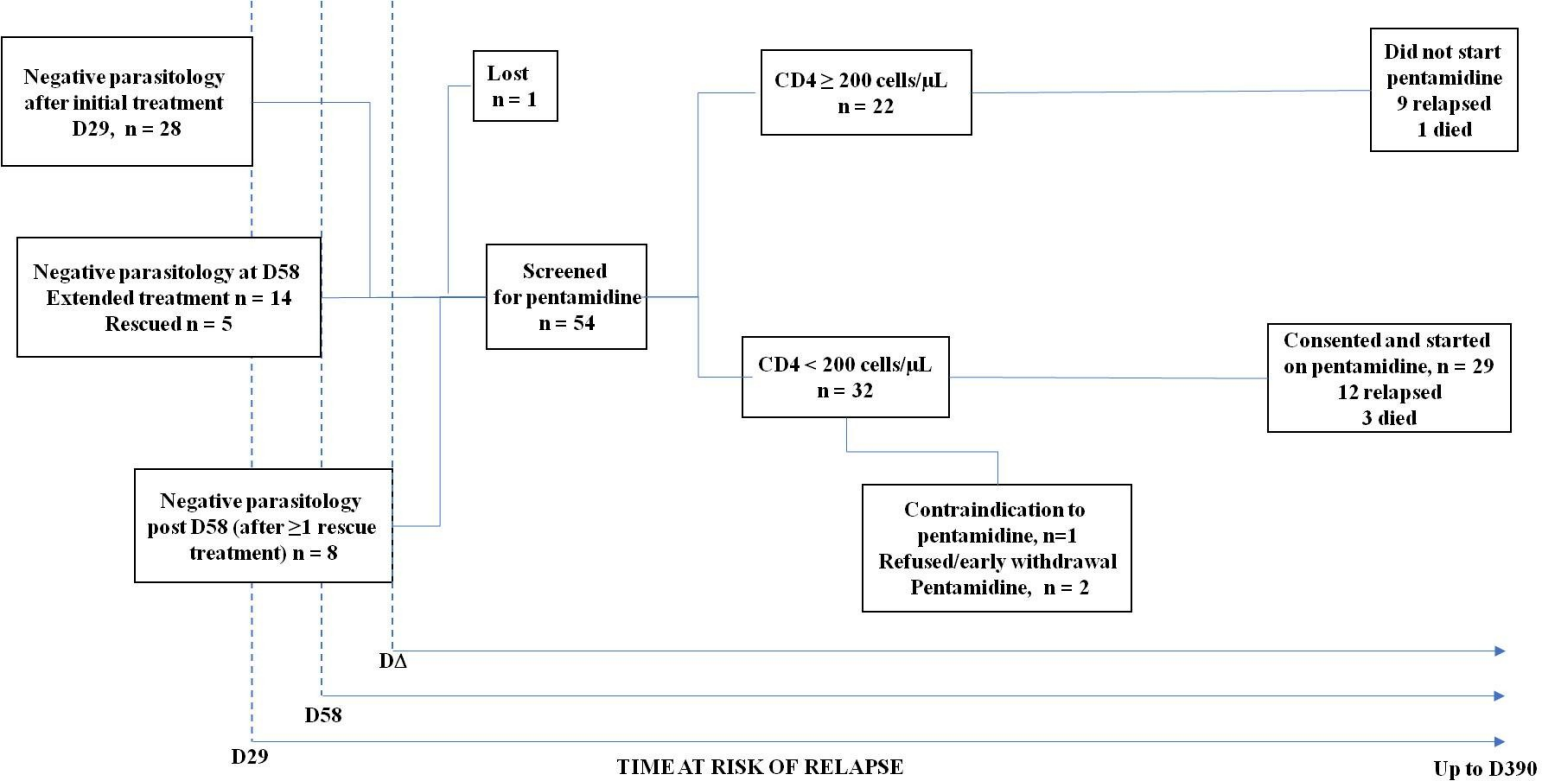
Follow-up period flow diagram. The maximum follow-up time was one year from initiation of VL treatment. However, patients only entered the cohort study on achieving negative parasitology. Hence, in general, patients had less than one year of follow-up in the cohort study.

Patients were enrolled from 14^th^ August 2014, and follow-up ended on 12^th^ August 2016. There were 55 patients who achieved parasitological cure with VL treatment(s) during the trial preceding this study. One patient was subsequently lost to follow-up and the remaining 54 patients were included in this cohort study (Fig 1).

### Sample size

This research aimed to study the long-term outcomes of VL patients with HIV co-infection enrolled in the above-mentioned clinical trial. The sample size of the clinical trial was determined based on expected efficacy of the initial treatment, and as such provides a fixed sample size for this cohort study as the number of patients surviving to negative parasitology post treatment for VL. There were no pre-specified sample size calculations specifically for the cohort study objectives reported here.

### Intervention

Patients who achieved parasitological cure but remained with a CD4 count below 200 cells/μL at the end of VL treatment were approached and consent was sought for pentamidine secondary prophylaxis. Patients with contraindications for pentamidine (renal impairment, diabetes, known hypersensitivity) were excluded from this intervention but were followed up as per their initial consent. Patients with CD4 cell counts above 200/μL were followed without secondary prophylaxis.

Pentamidine isethionate (Pentacarinat) secondary prophylactic treatment was started one month after the negative test of cure (completion of VL treatment). Every month, a dose of 4 mg/kg body weight of the salt reconstituted in 5 mL distilled water was re-diluted in 200 mL of 5% dextrose in saline or normal saline solution and infused over one hour with the patient in supine position. Patients were kept in the ward during administration and observed with frequent blood pressure monitoring for one hour before discharge. The blood glucose level was monitored prior to each infusion, and other metabolic panel tests (blood sugar level, renal function, liver function, serum electrolytes) were run every 6 months.

Patients were offered continued ART, co-trimoxazole prophylaxis and adherence counselling. Follow-up arrangements for HIV care were made at ART clinics. Blood samples for HIV viral load were sent to a regional reference laboratory every six months. Arrangements for ART regimen changes were made when indicated in communication with the respective ART clinics.

### Follow-up

Follow-up started on the date negative parasitology was achieved and ended 390 days (D390) after the initiation of the first VL treatment in the preceding trial. This means that those patients requiring more than one course of treatment to clear parasites during their VL treatment, and therefore achieving negative parasitology later, were followed up for less time than those who responded to one course of VL treatment (Fig 1). In particular, such patients would typically have less than one year of follow up in the cohort study.

Patients eligible for pentamidine treatment were seen monthly for prophylactic treatment. There were two pre-specified follow-up time points for all patients, regardless of pentamidine prophylaxis; at 6 and 12 months after initiation of treatment for the current VL episode to check on long-term outcomes. They could present for unscheduled assessments as needed during the follow-up period (e.g. relapse, intercurrent diseases, serious adverse events (SAE)). All SAEs, drug related adverse events (AEs) and any event that could lead to pentamidine interruption were documented during the follow-up period but other non-serious AEs were not systematically captured.

### Outcomes

Relapse-free survival by D390 was specified in the protocol as the primary outcome of this cohort study. Time at risk was defined as the time from negative parasitology following VL treatment to the earliest of the following events: i) death, ii) relapse, iii) date of the D390 visit, and iv) date last seen (in case of loss to follow-up). Relapse-free survival was defined as reaching the end of the time at risk having neither died nor relapsed. Death (i) or relapse confirmed by positive parasitology (ii) correspond to a “failure” outcome. Conversely, alive and relapse-free at D390 (iii) or lost to follow up (iv) correspond to censoring.

### Statistical methods

Relapse-free survival at one year was estimated using Kaplan-Meier methods to account for variable time at risk in all patients and within two sub-groups; i) CD4 ≥200 cells/μL not receiving pentamidine secondary prophylaxis and ii) CD4<200 cells/μL receiving pentamidine secondary prophylaxis. Although these groups were not defined a priori, risk factors were found to differ substantially between them. Graphical presentation of Kaplan-Meier analyses shows probability of failure rather than survival to illustrate timing of relapses and deaths in this cohort.

Poisson regression was used to investigate univariable risk factors associated with relapse or death, again accounting for variable time at risk. This analysis was performed within the two sub-groups mentioned above. A multivariable model was built including those factors found to be associated with relapse or death in univariable analyses, i.e. with 95% confidence intervals (CIs) excluding the null effect of 1. In principle a single model could be fitted to both subgroups by including interactions between subgroup and the risk factors. In practice, however, this was not possible due to small cell frequencies.

Compliance to pentamidine in those who were eligible was calculated as the percentage of patients who received all monthly treatments for which they were eligible, i.e. until relapse, death or D390 in surviving relapse-free patients.

Safety analyses comprised of summarizing the proportion of patients experiencing SAEs, an SAE related to pentamidine, and an AE that required discontinuation of pentamidine treatment. AE is defined as any untoward medical occurrence (any unfavourable and unintended sign, symptom or disease, including an abnormal laboratory finding) in temporal association with the use of the investigational treatment i.e. after the start of pentamidine. Causality relation to pentamidine is based on the known AEs listed in the Summary of Product Characteristics available from the manufacturer. Grading of the severity of the events was based on Common Terminology Criteria for Adverse Events (CTCAE), Version 4.0 [17]. For events not described in CTCAE, severity of AE is graded as mild (symptoms that did not require additional treatment), moderate (symptoms that require additional treatment and get controlled), severe (symptoms that require multiple treatments and may not resolve despite treatment). The definition of SAE was according to ICH-GCP guidelines (life threatening events or events that led to disability, hospitalization, death, or congenital anomalies).

### Ethical statement

The research protocol was approved by the Ethiopian regulatory authority (Food, Medicine, Health Care Administration and Control Authority, FMHACA), the National Research Ethics Review Committee (NRERC), the Institutional Review Board of the University of Gondar in Ethiopia, the Ethics Review Board of Médecins Sans Frontières, the London School of Hygiene and Tropical Medicine Research Ethics Committee, the Antwerp University Hospital Ethics Committee, and the Institute of Tropical Medicine, Antwerp Institutional Review Board. All patients were >18years old and were included into the study after written informed consent was given. Patients received all the required treatments free of charge including treatments for adverse events and intercurrent diseases. Food and transport support were provided.

## Results

### Patient description

Except for one female, all the patients were male migrant workers with a median age of 33 years. Among the 54 patients, 28 (52%) were relapse VL cases, and 27 (50%) were malnourished with body mass index (BMI) < 18.5kg/m^2^. Most of the patients, 39 (72%), were already on antiretroviral treatment (ART) when VL was diagnosed with 27 (50%) being on ART for six months or more. About two-thirds had a high *Leishmania* parasite load (grade of +5 and +6) at VL diagnosis. Two-thirds of the patients had previously been treated with the Ambisome+miltefosine combination. Overall, 50% required more than one treatment course (Table 1). More than one course of treatment was required for 67% of patients on Ambisome monotherapy and 40% of patients on the combination regimen. Patients who required more than one treatment course received the same treatment twice, or at least one course of rescue treatment (Table 2).

**Table 1:** Characteristics of patients achieving negative parasitology at end of VL treatment and assessed for eligibility to receive pentamidine.

		CD4 < 200 cells/μL and started pentamidine	CD4 ≥ 200 cells/μL	CD4<200 cells/μL who did not start pentamidine^a^	Overall
		N = 29	N = 22	N = 3	N = 54
Site	Gondar	17 (59)	11 (50)	1	29 (54)
	Abdurafi	12 (41)	11 (50)	2	25 (46)
Sex	Female	1 (3)	0 (0)	0	1 (2)
	Male	28 (97)	22 (100)	3	53 (98)
Age, years	Median (IQR)	33 (27–51)	32.5 (21–46)	37 (28–42)	33 (21–51)
Relapse status^b^	Primary	13 (45)	11 (50)	2	26 (48)
	Relapse	16 (55)	11 (50)	1	28 (52)
Parasite count^c^	1+ to 4+	9 (31)	6 (27)	2	17 (31)
	5+ to 6+	20 (69)	15 (68)	0	35 (65)
Anti-retroviral treatment at VL diagnosis^d^	No, or for <6 months	15 (52)	9 (41)	2	26 (48)
	Receiving ART for ≥6 months	13 (45)	13 (59)	1	27 (50)
VL treatment^e^	Ambisome	10 (34)	7 (32)	1	18 (33)
	Ambisome+Miltefosine	19 (66)	15 (68)	2	36 (67)
Number of courses of VL treatment^f^	1	18 (62)	7 (32)	2	27 (50)
	>1	11 (38)	15 (68)	1	27 (50)
BMI at negative parasitology	< 18.5 kg/m2	15 (52)	10 (45)	2	27 (50)
	≥ 18.5 kg/m2	14 (48)	12 (55)	1	27 (50)
CD4 count at negative parasitology (cells/μL)	<50	4 (14)	0 (0)	0	4 (7)
	50–99	6 (21)	0 (0)	2	8 (15)
	100 to 199	19 (66)	0 (0)	1	20 (37)
	200–349	0 (0)	12 (55)	0	12 (22)
	≥350	0 (0)	10 (45)	0	10 (19)
	Median (IQR)	110 (76–151)	337 (282–425)	90 (84–146)	173 (106–305)

Abbreviations: BMI, Body mass index; IQR, Interquartile range; VL, visceral leishmaniasis

Data are n (%) unless otherwise indicated

^a^Three patients with CD4 count <200cells/μL did not start pentamidine because of contraindication, refusal and early withdrawal

^b, c^ At the time of presentation for treatment of VL episode and randomization in the previous trial; one missing value for baseline parasite count.

^d^One missing value, lacking the ART start date

^e^ Treatment allocation as part of previous trial, although depending on parasitological and clinical improvement at end of first treatment regimen, patients may have received no further treatment, another course of the same treatment, and/or one or more courses of rescue medication.

^f^One course of treatment refers to those who had parasitological cure by D29, and ≥2 refers to those who required treatment for 58 days or more, either as a repeat course of the same treatment and, or one or more courses of rescue treatment.

**Table 2:** Summary table of pentamidine initiation, compliance and safety based on patient type for patients who started pentamidine.

	Patient status at baseline (D0)		
Number (%) of patients	Primary VL	Relapse VL	Total
	N = 13	N = 16	N = 29
Timing of negative parasitology:			
D29 (eligible for 12 treatments)	7 (54)	11 (69)	18 (62)
D58 (eligible for 11 treatments)	3 (23)	3 (19)	6 (21)
PostD58 (eligible for <11 treatments)	3 (23)	2 (13)	5 (17)
100% compliance^a^, n (%)	10 (77)	12 (75)	22 (76)
Experienced serious ADR, n (%)^b^	1 (8)	0 (0)	1 (3)
Pentamidine stopped due to ADR	0 (0)	0 (0)	0 (0)

Abbreviations: ADR–adverse drug reaction; VL–visceral leishmaniasis

^a^Compliance was calculated as the percentage of pentamidine treatments received out of the number of possible pentamidine treatments that could be given before relapse, death or the end of the study.

^b^Renal failure occurred in one patient who developed severe infection during follow up with monthly pentamidine which may be of multifactorial etiology.

Of the 54 patients who were followed up, 22 had a CD4 count ≥200 cells/μL at the time of achieving negative parasitology. Of the 32 with CD4 count <200 cells/μL, 29 were started on pentamidine prophylaxis (Table 1). The other three had a contraindication, refused to participate in the prophylaxis or withdrew before the first pentamidine infusion, and are not included in the analysis of relapse-free survival or risk factors for relapse or death (Fig 1). One of them required multiple VL treatments and relapsed around 4 months after parasitological cure. The other two patients were followed up for 9 months and 12 months, respectively, without relapse.

The type of VL (primary or relapse), *Leishmania* parasite load, BMI, ART status and duration and the VL treatment regimen used were comparable between those with a CD4 cell count <200/μL who started pentamidine, and those with a CD4 cell count ≥200/μL who did not receive pentamidine (Table 1). Of those with low CD4 at the end of VL treatment (<200 cells/μL), 62% of patients had received only one course of VL treatment, whereas in those with higher CD4 (≥200 cells/μL), it was lower at 32%.

The percentage of patients who received all pentamidine treatments for which they were eligible was 76% (Table 2).

### Relapse-free survival

**Fig 2:**
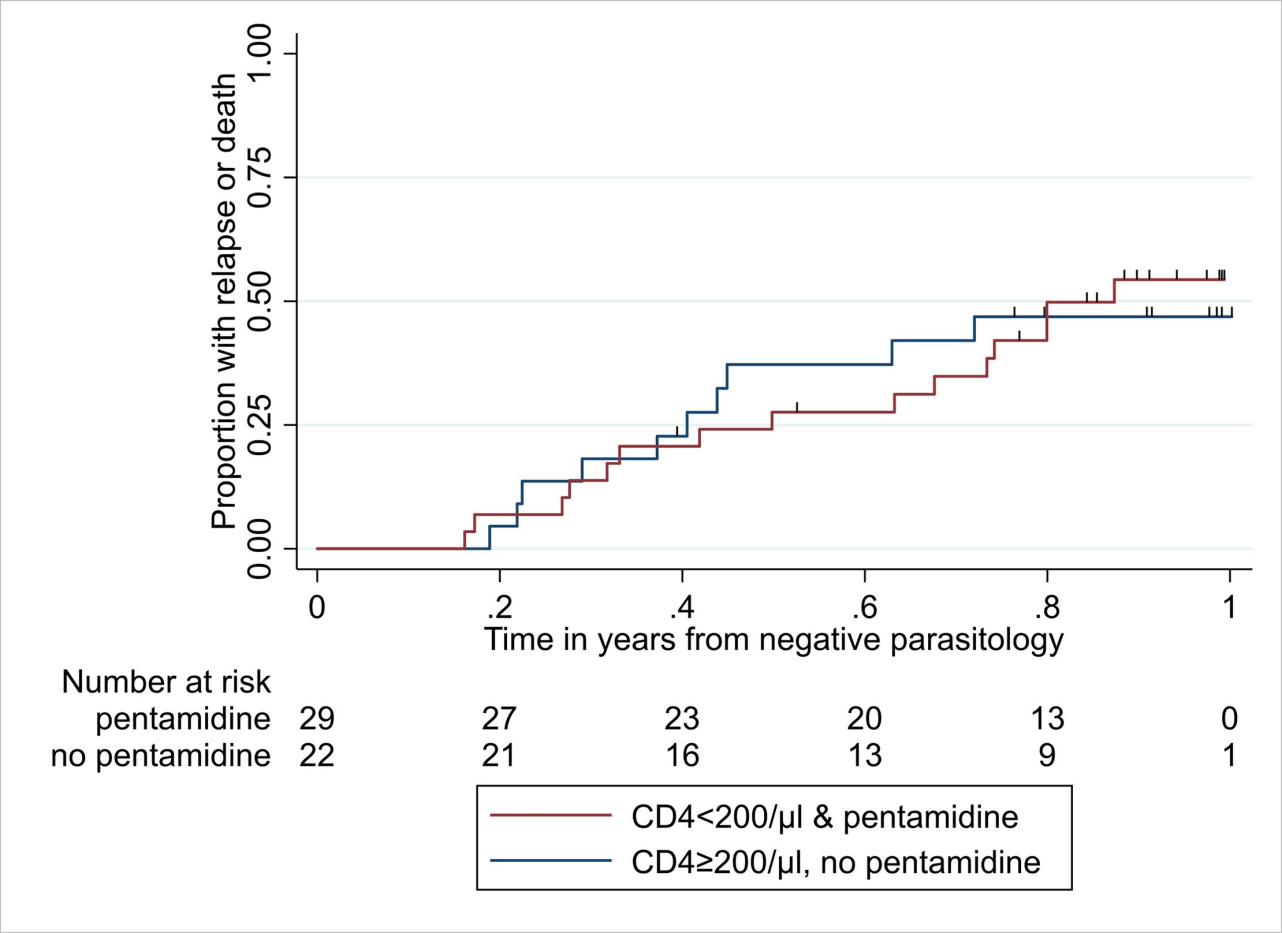
**Kaplan-Meier curve for relapse or death, for the two groups shown in Table 3: a) CD4 <200 cells/μL cells and pentamidine; and b) CD4 ≥200 cells/μL cells without pentamidine.** Overall, the median follow-up time was 0.77 years (9.2 months, range: 0.16–1.00 years). Median (range) follow-up time; CD4 <200 cells/**μ**L & pentamidine: 0.80 years (9.6 months, 0.16–0.99 years); CD4 ≥200 cells/**μ**L, no pentamidine: 0.74 years (8.9 months, 0.19–1.00 years). Thus, by one year all but one of the patients had either relapsed (n = 21), died (n = 4), or been censored (n = 25). The short vertical lines above each of the Kaplan-Meier curves shows the times at which censoring occurred. A large proportion of patients were censored because the scheduled time of follow-up in the cohort was 390 days minus the time between initiation of VL treatment and achieving negative parasitology (see ‘Follow-up’ subsection of Methods). This figure shows proportions while the regression analysis in Table 4 is based on rates per person-year. The numerical values of the proportions differ from those of the rates, but the approaches are consistent and complementary, as explained in footnote g of Table 4.

The Kaplan-Meier estimates of the percentage of all patients with relapse-free survival at one year was 50% (95%CI: 35–63%); 53% (95%CI: 30–71%) in the 22 patients with CD4 ≥200 cells/μL and 46% (95%CI: 26–63%) in the 29 patients with CD4<200 cells/μL who started pentamidine (Fig 2). The endpoint was reached because of death in one patient in the CD4 ≥200 cells/μL group. In the group with CD4 <200 cells/μL and on pentamidine, the endpoint was reached because of death in three patients, and two further patients died after reaching the endpoint due to VL relapse. If the events of interest are restricted to relapses, with pre-relapse deaths being considered as censored, then 57% (95%CI: 33–75%) of the 22 patients with CD4 ≥200 cells/μL, and 54% (95%CI: 33–71%) of the 29 patients with CD4 <200 cells/μL who started pentamidine were relapse-free at one year.

Pentamidine treatment was not interrupted or stopped due to any adverse drug reaction. One expected SAE was reported: renal failure, considered possibly related to pentamidine that led to death. This patient was also experiencing pyelonephritis, sepsis, VL relapse and multiple myeloma which might have contributed to the renal failure, either directly or by toxicity of the concomitant medications (Table 3).

**Table 3:** Serious adverse events occurring during follow-up after negative parasitology.

MedDRA preferred term	Intensity	Exposed to pentamidine	Onset^a^	Outcome	Possible relation to pentamidine
Strongyloidiasis	Death	Yes	32	Death	No
Septic shock	Death	No	144	Death	No
Cerebral toxoplasmosis	Life threatening	Yes	207	Resolved	No
Plasma cell myeloma^b^ Renal failure acute^b^	Death Death	Yes Yes	256 256	Death Death	No Yes
Retroviral infection	Death	Yes	270	Death	No
Splenic haemorrhage	Life threatening	Yes	275	Resolved	No
Hepatitis cholestatic	Life threatening	Yes	303	Resolved	No
Septic shock	Death	Yes	317	Death	No
Sepsis	Death	Yes	355	Death	No

^a^Time in days after negative parasitology

^b^Two serious adverse event reports in the same patient

### Factors associated with relapse or death: CD4 <200 cells/μL and on pentamidine

Among patients with low CD4 counts (<200 cells/μL) at the time of VL cure and who started pentamidine prophylaxis, no statistically significant risk factors for relapse or death were identified (Table 4).

**Table 4:** Associations with relapse or death by strata of CD4 count and pentamidine treatment.

		N	Time at risk (person-years)	Relapse or death (n)	Rate of relapse or death per person-years (95% CI)^g^	Unadjusted rate ratio (95% CI)	Adjusted rate ratio (95% CI)^h^
**CD4<200 cells/**μ**L & pentamidine (N = 29**^a^**) overall**		29	20.2	15	0.74 (0.45, 1–23)		
VL treatment before negative parasitology (trial arm)	Ambisome	10	8.0	3	0.37 (0.12, 1.16)	1	
	Ambisome + miltefosine	19	12.1	12	0.99 (0.56, 1.74)	2.64 (0.75, 9.36)	
Number of course of treatment required	1	18	12.6	9	0.72 (0.37, 1.38)	1	
	>1	11	7.61	6	0.79 (0.35, 1.75)	1.10 (0.39, 3.10)	
Relapse status (at randomization)	Primary VL	13	9.65	5	0.52 (0.22, 1.24)	1	
	Relapse VL	16	10.5	10	0.95 (0.51, 1.77)	1.83 (0.63, 5.37)	
BMI at time of negative parasitology	<18.5	15	10.0	9	0.90 (0.47, 1.72)	1	
	≥18.5	14	10.1	6	0.59 (0.27, 1.32)	0.66 (0.23, 1.85)	
ART at VL diagnosis^b^	Not yet receiving ART, or for <6months	15	10.6	8	0.76 (0.38, 1.52)	1	
	Receiving ART for ≥6months	13	9.3	6	0.65 (0.30, 1.44)	0.85 (0.30, 2.45)	
Baseline parasite count	1+ to 4+	9	7.0	3	0.43 (0.14, 1.33)	1	
	5+ to 6+	20	13.2	12	0.91 (0.52, 1.60)	2.11 (0.60, 7.49)	
**CD4≥200cells/μL & no pentamidine (N = 22**^c^**)overall**		22	14.6	10	0.69 (0.37, 1.28)		
VL treatment before negative parasitology (trial arm)	Ambisome^d^	7	2.8	6	2.17 (0.97, 4.82)	1	1
	Ambisome+miltefosine	15	11.8	4	0.34 (0.13, 0.90)	**0.16 (0.04, 0.55)**	0.34 (0.07, 1.72)
Number of treatment courses required	1^e^	7	6.33	0	0 (-, -)		
	>1	15	8.24	10	1.21 (0.65, 2.26)	-	
Relapse status (at randomization)	Primary	11	8.33	2	0.24 (0.06, 0.96)	1	1
	Relapse	11	6.23	8	1.28 (0.64, 2.57)	**5.35 (1.14, 25.2)**	**5.42 (1.14, 25.8)**
BMI at time of negative parasitology	<18.5	10	8.43	2	0.24 (0.06, 0.95)	1	1
	≥18.5	12	6.14	8	1.30 (0.65, 2.61)	**5.50 (1.17, 25.9)**	3.18 (0.44, 22.7)
ART at VL diagnosis	Not yet receiving ART, or for <6m	9	5.32	4	0.75 (0.28, 2.00)	1	
	Receiving ART for ≥6m	13	9.25	6	0.65 (0.29, 1.44)	0.86 (0.24, 3.05)	
Baseline parasite count^f^	1+ to 4+	6	4.4	1	0.23 (0.03, 1.61)	1	
	5+ to 6+	15	9.2	9	0.98 (0.51, 1.89)	4.34 (0.55, 34.3)	

Abbreviations: ART, Antiretroviral therapy; BMI, Body mass index; CI, confidence interval; VL, visceral leishmaniasis

^a^Of whom 12 relapsed and 3 died (15 with the endpoint).

^b^One missing value, lacking the ART start date

^c^Of whom 9 relapsed and 1 died (10 with the endpoint).

^d^All these patients required more than one course of treatment (either a repeat regimen of the same treatment or rescue)

^e^All who cleared parasites after one course of treatment were on Ambisome+Miltefosine

^f^One missing value

^g^Expressed as rates per person-year. The numerical values of the rates differ from those of the proportions in Fig 2 but the approaches are consistent and complementary. For example, converting the rate here (0.74 per person-year) to a proportion surviving relapse-free at one year gives e^-0.74/year × 1 year^ = 0.48, very similar to the 0.46 from the Kaplan-Meier analysis in Fig 2.

^h^Adjusted RRs from a multivariable model that includes all factors associated with relapse or death in univariable analyses; after adjustment, only patient type remains associated with rate of relapse or death. No univariable associations for patients with CD4 <200 cells/**μ**l who started pentamidine

### Factors associated with relapse or death: CD4 ≥200 cells/μL without pentamidine

In univariable analyses of patients with higher CD4 counts (≥200 cells/μL) at the time of VL cure, higher rates of relapse or death were detected in relapse cases compared to primary cases, and in patients with normal BMI compared to low BMI (<18.5kg/m^2^, Table 4). Patients previously treated with the combination regimen (Ambisome+miltefosine) for the VL episode had a lower rate of relapse or death, compared to those on Ambisome monotherapy (Table 4).

The relation between relapse-free survival and the number of treatments required to clear parasites was also investigated. Of note, among those with CD4 cell count above 200 cells/μl at the end of treatment, there were no relapses or deaths in the subgroup for whom one course of treatment was sufficient to clear parasites. Moreover, such patients had all been treated with the Ambisome+miltefosine combination. The number of relapses being zero prevents the calculation of a rate ratio for the ≥200 cells/μL group by the end of treatment. Conversely, all patients with CD4 ≥200 cells/μL at the time of negative parasitology who received Ambisome monotherapy had required more than one course of treatment to clear parasites.

In adjusted analyses, simultaneously accounting for the VL treatment regimen, relapse status (primary vs relapse) and BMI, only relapsed patients remained significantly associated with subsequent relapse or death (adjusted rate ratio (ARR) = 5.42, 95%CI: 1.1–25.8, Table 4).

## Discussion

The treatment of VL in HIV patients is complicated by a high rate of relapse in the first year after VL treatment [14,15]. Relapse leads to further immunosuppression, progression of HIV disease, predisposition to a number of opportunistic infections, failure of ART and death. Thus, it is important to comprehensively manage VL in HIV patients to ensure an effective initial treatment that is complemented by subsequent relapse prevention.

Long-term outcomes of VL in HIV patients are described in this prospective cohort study, with the use of pentamidine secondary prophylaxis for those with CD4 cell counts <200/μL. Parasitologically cured VL patients with HIV co-infection were followed up for one year. The study was conducted in Northwest Ethiopia which is one of the highest HIV-VL co-infection regions of the world. The population is largely young adult males, with relapsed VL status and malnutrition accounting for about half of the cases respectively and patients at different time period on ART. The results are likely to be generalizable to the rest of Ethiopia and East Africa. The management of patients in this cohort included offering ART to all and secondary prophylaxis to those with a CD4 count <200 cells/μL after achieving parasitological cure of VL. Three-quarters of the patients had 100% compliance for the monthly pentamidine infusions. Low CD4 count is a known risk factor for relapse of VL [15,16]. Despite this care package, several VL relapses occurred, regardless of CD4 count at the end of VL treatment. This indicates the possibility of other factors influencing the long-term outcomes. The one death that was possibly related to pentamidine was due to acute renal failure in a patient with multiple co-existing diseases that can affect renal status. The strength of the study was that, as a continuation of a clinical trial, it complied with GCP, had substantial resources for intensive follow up, which helped ensure few missing data and consequently reduced bias.

The probability of relapse-free survival at one year was 50% (95% CI: 35–63%) in all patients. One limitation of this study was that it was not adequately powered to test differences between the two sub-groups; CD4 ≥200 cells/μL without pentamidine versus CD4 <200 cells/μL and receiving pentamidine prophylaxis. Another limitation was that the allocation of pentamidine was dependent on CD4 count which complicates the interpretation of the results and does not allow for unbiased estimation of an overall effect of pentamidine prophylaxis, or an overall effect of CD4 count. Interestingly, comparable probabilities of relapse-free survival were seen in the two groups, 53% (95%CI 30–71%) and 46% (95%CI 26–63%) respectively. A direct comparison of the two groups (patients with CD4<200 given prophylaxis and patients with CD4>200 without prophylaxis) based on a hypothesis of a difference was not included in our study as we may not expect a difference between these two groups if pentamidine can reduce the risk of relapse or death due to low CD4, bringing the risk more in line with risk in those with higher CD4 and not on pentamidine, and we propose this as a hypothesis. According to previous reports, 60–70% of VL cases with HIV co-infection and on ART relapse within one year in the absence of secondary prophylaxis, the relapse rate being higher with a lower CD4 count [14,15]. The few existing reports on the use of secondary prophylaxis showed a relapse-free survival rate at one year ranging from 40–80% and a relapse rate among patients without secondary prophylaxis of 50–100% [11,18–21]. Most of these studies were case series studies from a region with *L*. *infantum* transmission, using different drugs and different follow-up patterns.

In a recently conducted interventional cohort at the same study sites in Ethiopia, primary, relapse and past VL cases were enrolled. Pentamidine was given to those who achieved negative parasitology for the VL episode if their CD4 cell count was <200 cells/μL, and to all relapse cases regardless of CD4 count. The relapse-free survival rate at one year in all patients was 71% [16]. Looking more in depth at subgroups, the relapse-free survival rate for patients with CD4 <200 cells/μL (n = 45) in this previous study was 68% (95%CI: 52%-80%) and in those with CD4 ≥200 cells/μL (n = 12), it was 82% (95%CI: 45%-95%), with up to 12 months of pentamidine prophylaxis. There was no apparent difference in relapse-free survival observed in the groups with CD4 <200 cells/μL between the two studies, based on the overlap between the 95% confidence intervals around relapse-free survival in each study.

The potential benefit of pentamidine amongst patients in the current study can be hypothesized to result from pentamidine treatment reducing the rate of relapse amongst those more likely to otherwise do so, to a rate closer to that observed in patients with a higher CD4 count. Although the use of secondary prophylaxis reduced the risk of relapse, there was still a notably high number of patients who continued to relapse in both groups (CD4 <200 and CD4 ≥200 cells/μL). There are earlier reports showing relapse at higher CD4 count and also with secondary prophylaxis use [6,11].

Multiple factors may play a role in the risk of VL relapse. In this study, CD4 count, duration on ART, VL relapse status (primary vs relapse), the *Leishmania* parasite load at diagnosis, the antileishmanial treatment regimen (Ambisome monotherapy vs Ambisome+miltefosine combination), the duration of VL treatment and BMI were evaluated. In the group of patients with CD4 cell count <200/μL and receiving pentamidine, no additional independent risk factors were found to be associated with an increased rate of relapse. There is a need for additional, adequately powered studies to assess the risk factors for persistent immunosuppression in these patients. VL disease while on ART and low CD4 count is a WHO AIDS-defining illness and an indicator of advanced HIV disease and possibly undiagnosed ART failure [22,23]. For patients with profound immunodeficiency, ART, anti-*Leishmania* combination therapy and secondary prophylaxis seem insufficient to prevent relapse. The high relapse rate among these groups of patients indicates the need to explore other treatment modalities. These can be more frequent and/or higher doses of the prophylaxis or other interventions that can rapidly improve immunity [24]. Serum level of pentamidine was not checked in the study and the optimal prophylaxis dose is not known. Due to the differences in the formulation of pentamidine, the dose used in this study might be lower [25]. Beyond that, the goal should be for early detection and management of VL before profound immune deficiency sets in [26].

In the group of patients who had a CD4 cell count above 200/μl at the end of VL treatment (stable on ART), past history of VL treatment (relapse) was an independent risk factor for subsequent relapse, as has previously been observed [15,27]. The current study reconfirms that patients with a previous history of relapse have a higher risk of relapse regardless of their CD4 cell count, adjusted rate ratio of 5.42 (1.14, 25.8). The small sample size in this group did not allow for further breakdown of the CD4 level and subgroup analysis. However, the findings indicate that CD4 level recommendation for secondary prophylaxis has to be re-visited and higher cut-off values be recommended [28].

Although the study was not adequately powered for a pre-specified magnitude of rate ratio, it is plausible to hypothesize a worse prognosis for patients whose VL episodes were hard-to-treat. Hard-to-treat patients are likely to include those who develop VL while on ART for more than 6 months, have a persistently low CD4 count (<200 cells/μL) after VL treatment (advanced HIV disease), those who require longer treatment to cure VL, those with treatment failure for a combination regimen and previous history of VL treatment [15]. Because CD4 recovery can take time, those who received longer VL treatment (negative parasitology at or after D58) were likely to achieve CD4 cell counts above 200/μL and thus become ineligible for secondary prophylaxis in this study, while they still fall in the category of hard-to-treat patients. It could be hypothesized that those patients would have benefited from the secondary prophylaxis, hence avoiding some of the observed relapses in the group with the higher CD4 cell count. In this study 50% of those with CD4 cell count ≥200cells/μl were relapsed patients who might have benefited from secondary prophylaxis.

Patients who require prolonged initial VL treatment (>1 month) might be those with advanced disease and high initial parasite load, or those receiving less effective initial treatment (e.g. Ambisome monotherapy). A highly effective initial VL treatment regimen (e.g. combination therapy) seems a more favorable approach, especially for hard-to-treat VL patients.

Likewise, faster parasitological cure of VL and CD4 recovery ≥200 cells/μL (cure by D29) can be a sign of mild disease and relatively well-preserved immunity. Of the seven patients who responded to initial treatment (parasite clearance) by D29 and had CD4 count ≥200 cells/μL (thus without pentamidine prophylaxis), none relapsed or died. Although based on a very small number of patients, this could suggest that secondary prophylaxis may not be a priority for patients who respond to one course of treatment and achieve a CD4 level ≥200 cells/μL. This might be related to early diagnosis of VL in ART stable patients. On the other hand, the decision for secondary prophylaxis may need to be based on the CD4 level at the time of VL diagnosis rather than after VL treatment.

In general, we have observed that the long-term outcome of VL in HIV patients is affected by multiple factors–importantly the level of immunity, history of VL treatment and the use of secondary prophylaxis. While a clear trend of benefit from the secondary prophylaxis is observed among those with low immunity, it should be noted that the treatment of these patients requires a multifactorial approach. Effective ART is a crucial component. Although the management of HIV included ART provision, clinical and CD4 cell monitoring; regular viral load determination was not possible due to the limited services available in the country during the study period and the delayed provision of results. The available data suggest that not all patients were on successful ART treatment and only a few had second line ART (S1, S2 and S3 Tables). Sustainable treatment of HIV-related opportunistic infection without effective ART is impossible. Integration of HIV treatment within the treatment programs of endemic opportunistic diseases is important for effective disease control [8,29]. HIV viral load results are important for patient management decisions and facilities in high endemic regions need to be upgraded with such services.

In conclusion, the VL relapse rate in HIV co-infected patients is high irrespective of CD4 level. Secondary prophylaxis with pentamidine was found to be safe except in patients with risk factors for renal failure and could help prolong the disease-free survival of those with a CD4 count below 200cells/μL to a rate comparable to that for patients with CD4 count above 200 cells/μL and not receiving secondary prophylaxis. There are studies supporting its effectiveness and safety from both *L*. *donovani* and *L*. *infantum* regions [12,16,30,31]. However, the available data to date are based on small numbers of patients and from non-randomized studies, and are therefore below the ideal level of evidence needed to recommend implementation. Taking into consideration the high mortality and morbidity of VL-HIV co-infection and the urgent need for better management, we strongly recommend the use of secondary prophylaxis as an integral part of VL management in HIV. Priority cases for secondary prophylaxis are patients whose CD4 cell count remain <200/μL after effective VL treatment and those with a history of VL treatment (VL relapses). A monthly infusion of pentamidine is a suitable option in terms of feasibility and safety, except for patients with renal diseases. Future prospective research studies could investigate alternative prophylactic regimens, different dosing and frequency to improve relapse-free survival, alongside new treatment approaches for hard-to-treat patients.

## Supporting information

S1 Checklist STROBE checklist. (DOCX)

S1 Table HIV viral Loads (copies/ml) of patients on day 0, day 210 and day 390 of their follow up starting from the VL diagnosis. (DOCX)

S2 Table Summary of CD4 count in categories, HIV viral load and anteretroviral regimens at baseline with the visceral leishmaniasis treatment used. (DOCX)

S3 Table The pattern of CD4 cell count and HIV viral load during follow up. (DOCX)
