# Supplementary material for: Long term outcomes and prognostics of visceral leishmaniasis in HIV infected patients with use of pentamidine as secondary prophylaxis based on CD4 level: a prospective cohort study in Ethiopia
Source: PLoS Negl Trop Dis. 2019 Feb 21;13(2):e0007132. doi: 10.1371/journal.pntd.0007132 (PMC6400407; doi:10.1371/journal.pntd.0007132)
Supplement: S1 Checklist — (DOCX) [file pntd.0007132.s001.docx]

STROBE Statement: Pentamidine cohort

|  | Item No | Recommendations | Section |
| --- | --- | --- | --- |
| Title and abstract | 1 | (a) Indicate the study’s design with a commonly used term in the title or the abstract | The title specifies a cohort design. |
|  |  | (b) Provide in the abstract an informative and balanced summary of what was done and what was found | A structured abstract is provided. |
| **Introduction** |  |  |  |
| Background/rationale | 2 | Explain the scientific background and rationale for the investigation being reported | The Introduction section. |
| Objectives | 3 | State specific objectives, including any prespecified hypotheses | Penultimate section of the Introduction section. |
| **Methods** |  |  |  |
| Study design | 4 | Present key elements of study design early in the paper | ‘Study Design’ subsection of the Methods section. |
| Setting | 5 | Describe the setting, locations, and relevant dates, including periods of recruitment, exposure, follow-up, and data collection | ‘Setting’ and ‘Participants’ subsections of the Methods section. |
|  | 6 | (a) *Cohort study*—Give the eligibility criteria, and the sources and methods of selection of participants. Describe methods of follow-up | ‘Setting’ and ‘Follow up’ subsections of the Methods section. |
|  |  | (b) *Cohort study*—For matched studies, give matching criteria and number of exposed and unexposed | Not applicable |
| Variables | 7 | Clearly define all outcomes, exposures, predictors, potential confounders, and effect modifiers. Give diagnostic criteria, if applicable | ‘Intervention’ and ‘Outcomes’ subsections of the Methods section. |
| Data sources/ measurement | 8* | For each variable of interest, give sources of data and details of methods of assessment (measurement). Describe comparability of assessment methods if there is more than one group | ‘Intervention’, ‘Follow up’ and ‘Outcomes’ subsections of the Methods section. |
| Bias | 9 | Describe any efforts to address potential sources of bias | “Statistical methods section of “Methods” and Second paragraph of the Discussion section. |
| Study size | 10 | Explain how the study size was arrived at | ‘Sample size’ subsection of the Methods section. |
| Quantitative variables | 11 | Explain how quantitative variables were handled in the analyses. If applicable, describe which groupings were chosen and why | Groupings such as those for CD4 were chosen to be in accordance with the HIV literature. |
| Statistical methods | 12 | (a) Describe all statistical methods, including those used to control for confounding | ‘Statistical methods’ subsection of the Methods section. |
|  |  | (b) Describe any methods used to examine subgroups and interactions | ‘Statistical methods’ subsection of the Methods section, and ‘Patient description’ subsection of the Results section. |
|  |  | (c) Explain how missing data were addressed | The few missing data are described in the footnotes to Table 4. |
|  |  | (d) *Cohort study*—If applicable, explain how loss to follow-up was addressed | Losses to follow-up are addressed by treating them as censored in the survival analysis. |
|  |  | (e) Describe any sensitivity analyses | None. |
| **Results** |  |  |  |
| Participants | 13* | (a) Report numbers of individuals at each stage of study—eg numbers potentially eligible, examined for eligibility, confirmed eligible, included in the study, completing follow-up, and analysed | ‘Participants’ subsection of the Methods section, and ‘Number at risk’ table of Figure 2. |
|  |  | (b) Give reasons for non-participation at each stage | ‘Patient description’ subsection of the Results section. |
|  |  | (c) Consider use of a flow diagram | Not done, because of the complete enrolment of those eligible at the end of the previous trial. |
| Descriptive data | 14* | (a) Give characteristics of study participants (eg demographic, clinical, social) and information on exposures and potential confounders | Tables 1 & 2. |
|  |  | (b) Indicate number of participants with missing data for each variable of interest | Tables 1, 2 & 4. |
|  |  | (c) *Cohort study*—Summarise follow-up time (eg, average and total amount) | Figure 2. |
| Outcome data | 15* | *Cohort study*—Report numbers of outcome events or summary measures over time | Table 4 and Figure 2. |
|  |  | *~~Case-control study~~*~~—Report numbers in each exposure category, or summary measures of exposure~~ |  |
| Main results | 16 | (a) Give unadjusted estimates and, if applicable, confounder-adjusted estimates and their precision (eg, 95% confidence interval). Make clear which confounders were adjusted for and why they were included | ‘Relapse-free survival’ and subsequent subsections of the Results section, and Table 4. |
|  |  | (b) Report category boundaries when continuous variables were categorized | Tables 1 & 4. |
|  |  | (c) If relevant, consider translating estimates of relative risk into absolute risk for a meaningful time period | Results are quoted as both rates and proportions. |
| Other analyses | 17 | Report other analyses done—eg analyses of subgroups and interactions, and sensitivity analyses | Not done. |
| **Discussion** |  |  |  |
| Key results | 18 | Summarise key results with reference to study objectives | Third and subsequent paragraphs of the Discussion section. |
| Limitations | 19 | Discuss limitations of the study, taking into account sources of potential bias or imprecision. Discuss both direction and magnitude of any potential bias | Third and subsequent paragraphs of the Discussion section. |
| Interpretation | 20 | Give a cautious overall interpretation of results considering objectives, limitations, multiplicity of analyses, results from similar studies, and other relevant evidence | Fourth and subsequent paragraphs of the Discussion section. |
| Generalisability | 21 | Discuss the generalisability (external validity) of the study results | Second paragraph of the Discussion section. |
| **Other information** |  |  |  |
| Funding | 22 | Give the source of funding and the role of the funders for the present study and, if applicable, for the original study on which the present article is based | Provided in the relevant section of the online submission system. |
| *Give information separately for cases and controls in case-control studies and, if applicable, for exposed and unexposed groups in cohort and cross-sectional studies. | | | |
