# Supplementary material for: Long term outcomes and prognostics of visceral leishmaniasis in HIV infected patients with use of pentamidine as secondary prophylaxis based on CD4 level: a prospective cohort study in Ethiopia
Source: PLoS Negl Trop Dis. 2019 Feb 21;13(2):e0007132. doi: 10.1371/journal.pntd.0007132 (PMC6400407; doi:10.1371/journal.pntd.0007132)
Supplement: S1 Table — (DOCX) [file pntd.0007132.s002.docx]

Supplemental Table 1: HIV viral Loads (copies/ml) of patients on day 0, day 210 and day 390 of their follow up starting from the VL diagnosis

| **ID** | **Day 0** | **Day 210** | **Day 390** |
| --- | --- | --- | --- |
| 101 | 162 | Undetected | Results could not be traced |
| 102 | 1170 | 206432 | 197867 |
| 103 | 8331300 | Undetected | Undetected |
| 104 | 182436 | Undetected | 730326 |
| 105 | < 150 | Undetected | Results could not be traced |
| 106 | 18539 | 4130 | 6930 |
| 107 | Undetected | Undetected | Undetected |
| 108 | 63527 | 208771 | 55648 |
| 109 | Undetected | Undetected | Undetected |
| 110 | 544812 | Results could not be traced | Undetected |
| 111 | Not Done | Not Done | Not Done |
| 112 | 959068 | Undetected | 62018 |
| 113 | 1437 | Not Done | Not Done |
| 114 | Undetected | Undetected | Undetected |
| 115 | 758 | Undetected | Undetected |
| 201 | Undetected | Undetected | Undetected |
| 202 | Undetected | Undetected | Results could not be traced |
| 203 | 168244 | Undetected | 12716 |
| 204 | 2400336 | 124895 | 1522684 |
| 205 | 462695 | 138704 | 208771 |
| 206 | 926 | 400583 | 36073 |
| 207 | 1194983 | Not Done | Not Done |
| 208 | 18079 | 1512987 | 2573434 |
| 209 | 302860 | 1456085 | 257443 |
| 210 | Undetected | Undetected | Undetected |
| 212 | 201308 | Not Done | Not Done |
| 213 | 201308 | 1858 | < 150 |
| 214 | Undetected | 774 | Undetected |
| 215 | Undetected | < 20 | Undetected |
| 216 | 66103 | Not Done | 37671 |
| 217 | 779 | Undetected | Undetected |
| 301 | Results could not be traced | 173 | 99495 |
| 302 | Undetected | Undetected | Undetected |
| 303 | 631887 | Undetected | Undetected |
| 304 | 8541105 | Not Done | Not Done |
| 305 | Undetected | Undetected | Undetected |
| 306 | 480472 | Not Done | Not Done |
| 307 | 5858397 | Not Done | Not Done |
| 308 | 634560 | Not Done | Not Done |
| 309 | 3348920 | Results could not be traced | Results could not be traced |
| 310 | 1552151 | Not Done | Not Done |
| 311 | Undetected | Undetected | 82 |
| 312 | 12831 | < 150 | 2856 |
| 313 | Unknown | Undetected | Undetected |
| 401 | 418439 | 418439 | Not Done |
| 402 | Undetected | Undetected | 46517 |
| 403 | >10 million | Undetected | Undetected |
| 404 | Undetected | Undetected | < 150 |
| 405 | Results could not be traced | Undetected | Undetected |
| 406 | 869 | Undetected | Undetected |
| 407 | Undetected | Undetected | Undetected |
| 408 | 256316 | 8342 | 561861 |
| 409 | 284 | Undetected | Undetected |
| 410 | 3611203 | Not Done | Not Done |
| 411 | 404 | Not Done | Not Done |
| 412 | 1585489 | 6249196 | Undetected |
| 413 | 3035849 | Undetected | Undetected |
| 414 | Undetected | Undetected | Undetected |
| 415 | 23481 | 3405848 | Unknown |
