# Supplementary material for: Long term outcomes and prognostics of visceral leishmaniasis in HIV infected patients with use of pentamidine as secondary prophylaxis based on CD4 level: a prospective cohort study in Ethiopia
Source: PLoS Negl Trop Dis. 2019 Feb 21;13(2):e0007132. doi: 10.1371/journal.pntd.0007132 (PMC6400407; doi:10.1371/journal.pntd.0007132)
Supplement: S2 Table — (DOCX) [file pntd.0007132.s003.docx]

Supplemental table 2: Summary of CD4 count in categories, HIV viral load and anteretroviral regimens at baseline with the visceral leishmaniasis treatment used

|  | | **AmBisome®:** | | **AmBisome® + Miltefosine:** | |
| --- | --- | --- | --- | --- | --- |
|  |  | ART at baseline | | ART at baseline | |
|  |  | No | Yes | No | Yes |
| CD4 count (cells/µl) | <50 | 2 (33) | 6 (46) | 6 (55) | 11 (39) |
|  | 50-99 | 4 (67) | 2 (15) | 3 (27) | 11 (39) |
|  | 100 to 199 | 0 (0) | 5 (38) | 2 (18) | 5 (18) |
|  | 200-349 | 0 (0) | 0 (0) | 0 (0) | 1 (4) |
|  | ≥350 | 0 (0) | 0 (0) | 0 (0) | 0 (0) |
|  | Median (IQR) | 70 (30-77) | 50 (44-129) | 45 (33-97) | 56 (31-96) |
| Viral Load (copies per ml)^1^ | <150 – undetectable | 0 (0) | 7 (54) | 0 (0) | 9 (32) |
|  | 150 to <3 log_10_ | 0 (0) | 3 (23) | 0 (0) | 4 (14) |
|  | ≥3 to <4 log_10_ | 0 (0) | 0 (0) | 0 (0) | 2 (7) |
|  | ≥4 to <5 log_10_ | 0 (0) | 1 (8) | 1 (9) | 4 (14) |
|  | ≥5 to <6 log_10_ | 3 (50) | 2 (15) | 4 (36) | 4 (14) |
|  | ≥6 log_10_ | 3 (50) | 0 (0) | 5 (45) | 3 (11) |
|  | missing | 0 (0) | 0 (0) | 1 (9) | 2 (7) |
|  | Median (IQR)^1^ | 6.51 log_10_ (5.68 -6.92 log_10_) | 150  (150-926) | 5.98 log_10_ (5.30 -6.38 log_10_) | 869  (150-5.26 log_10_) |
| Regimen^2^ | ABC+3TC+EFV | 0 (0) | 0 (0) | 0 (0) | 1 (4) |
|  | ABC+3TC+LPV/r | 0 (0) | 0 (0) | 0 (0) | 1 (4) |
|  | ABC+DDI+LPV/r | 0 (0) | 0 (0) | 0 (0) | 1 (4) |
|  | AZT+3TC+EFV | 0 (0) | 0 (0) | 0 (0) | 2 (7) |
|  | AZT+3TC+NVP | 0 (0) | 1 (8) | 0 (0) | 4 (14) |
|  | TDF+3TC+EFV | 0 (0) | 12 (92) | 0 (0) | 18 (64) |
|  | TDF+3TC+NVP | 0 (0) | 0 (0) | 0 (0) | 1 (4) |
|  | none | 6 (100) | 0 (0) | 11 (100) | 0 (0) |

^1^ Values less than detection threshold, or less than 150 copies/ml have been set to 150 copies/ml.

^2^ ABC Abacavir, 3TC Lamivudine, AZT Zidovudine, LPV Lopinavir, r Ritonavir, EFV Efavirenz, NVP Nevirapine
