# Supplementary material for: Long term outcomes and prognostics of visceral leishmaniasis in HIV infected patients with use of pentamidine as secondary prophylaxis based on CD4 level: a prospective cohort study in Ethiopia
Source: PLoS Negl Trop Dis. 2019 Feb 21;13(2):e0007132. doi: 10.1371/journal.pntd.0007132 (PMC6400407; doi:10.1371/journal.pntd.0007132)
Supplement: S3 Table — (DOCX) [file pntd.0007132.s004.docx]

Supplemental table 3: The pattern of CD4 cell count and HIV viral load during follow up

|  |  | Baseline | | D210 | | | D390 | |
| --- | --- | --- | --- | --- | --- | --- | --- | --- |
|  |  | AmBisome® | AmBisome® + Miltefosine | | AmBisome® | AmBisome® + Miltefosine | AmBisome® | AmBisome® + Miltefosine |
| CD4 count (cells/µl) | <50 | 8 (42) | 17 (44) | | 1 (5) | 2 (5) | 1 (5) | 2 (5) |
|  | 50-99 | 6 (32) | 14 (36) | | 3 (16) | 3 (8) | 3 (16) | 1 (3) |
|  | 100 to 199 | 5 (26) | 7 (18) | | 6 (32) | 10 (26) | 7 (37) | 13 (33) |
|  | 200-349 | 0 (0) | 1 (3) | | 4 (21) | 9 (23) | 2 (11) | 9 (23) |
|  | ≥350 | 0 (0) | 0 (0) | | 2 (11) | 7 (18) | 2 (11) | 7 (18) |
|  | Missing | 0 (0) | 0 (0) | | 3 (16) | 8 (21) | 4 (21) | 7 (18) |
|  | Median (IQR) | 69  (30-121) | 54  (33-96) | | 150  (101-271) | 208  (120-321) | 151  (94-218) | 202  (119-335) |
| Viral Load (copies per ml)^1^ | <150 – undetectable | 7 (37) | 9 (23) | | 12 (63) | 18 (46) | 7 (37) | 19 (49) |
|  | 150 to <3 log_10_ | 3 (16) | 4 (10) | | 0 (0) | 2 (5) | 0 (0) | 0 (0) |
|  | ≥3 to <4 log_10_ | 0 (0) | 2 (5) | | 0 (0) | 3 (8) | 0 (0) | 2 (5) |
|  | ≥4 to <5 log_10_ | 1 (5) | 5 (13) | | 0 (0) | 0 (0) | 4 (21) | 3 (8) |
|  | ≥5 to <6 log_10_ | 5 (26) | 8 (21) | | 3 (16) | 3 (8) | 1 (5) | 4 (10) |
|  | ≥6 log_10_ | 3 (16) | 8 (21) | | 1 (5) | 3 (8) | 0 (0) | 2 (5) |
|  | Missing | 0 (0) | 3 (8) | | 3 (16) | 10 (26) | 7 (37) | 9 (23) |
|  | Median (IQR)^1^ | 926  (150-480,472) | 18,309  (150-634,560) | | 150  (150-104,461) | 150  (150-4,130) | 150  (150-36,073) | 150  (150-62,018) |

^1^ Values less than detection threshold, or less than 150 copies/ml have been set to 150 copies/ml.
